# We Need to Talk About Lung Ultrasound Score: Prediction of Intensive Care Unit Admission with Machine Learning

**DOI:** 10.3390/jimaging11020045

**Published:** 2025-02-07

**Authors:** Duarte Oliveira-Saraiva, João Leote, Filipe André Gonzalez, Nuno Cruz Garcia, Hugo Alexandre Ferreira

**Affiliations:** 1 Instituto de Biofísica e Engenharia Biomédica, Faculdade de Ciências da Universidade de Lisboa, 1749-016 Lisboa, Portugal; 2 LASIGE, Faculdade de Ciências da Universidade de Lisboa, 1749-016 Lisboa, Portugal; 3 Critical Department, Hospital Garcia de Orta E.P.E, 2805-267 Almada, Portugal

**Keywords:** COVID-19, ICU, lung ultrasound, lung ultrasound score, machine learning

## Abstract

The admission of COVID-19 patients to the Intensive Care Unit (ICU) is largely dependent on illness severity, yet no standard criteria exist for this decision. Here, lung ultrasound (LU) data, blood gas analysis (BGA), and clinical parameters from venous blood tests (VBTs) were used, along with machine-learning (ML) models to predict the need for ICU admission. Data from fifty-one COVID-19 patients, including ICU admission status, were collected. The information from LU was gathered through the identification of LU findings (LUFs): B-lines, irregular pleura, subpleural, and lobar consolidations. LU scores (LUSs) were computed by summing predefined weights assigned to each LUF, as reported in previous studies. In addition, individual LUFs were analyzed without calculating a total LUS. Support vector machine models were built, combining the available clinical data to predict ICU admissions. The application of ML models to individual LUFs outperformed standard LUS approaches reported in previous studies. Moreover, combining LU data with results from other medical exams improved the area under the receiver operating characteristic curve (AUC). The model with the best overall performance used variables from all three exams (BGA, LU, VBT), achieving an AUC of 95.5%. Overall, the results demonstrate the significant role of ML models in improving the prediction of ICU admission. Additionally, applying ML specifically to LUFs provided better results compared to traditional approaches that rely on traditional LUSs. The results of this paper are deployed on a web app.

## 1. Introduction

The intensive care unit (ICU) is a specialized department in the hospital that takes care of critically ill patients The admission of hospitalized COVID-19 patients to the ICU occurs in approximately 17 to 35% of cases due to respiratory failure [1]. The ratio of arterial oxygen partial pressure to fractional inspired oxygen (P/F) is a key indicator for assessing respiratory failure and assists clinicians in defining the need for ICU admission [1,2,3,4,5]. However, there are no accepted standard criteria in clinical practice to determine ICU admission, leaving a critical gap in the decision-making process.

To address this issue, various predictive models have been developed. Liam et al. [6] introduced the GRAM score to predict critical illness, i.e., ICU admission, need for invasive mechanical ventilation or death of COVID-19 patients. Although the GRAM score demonstrated good predictive ability (area under the receiver operating characteristic curve (AUC) = 88%), it relies on chest X-rays, laboratory parameters, and clinical history, which are not always readily available in emergency settings. Considering this, Boero et al. [7] proposed a prognostic score (COWS) incorporating lung ultrasound (LU), a faster and more accessible alternative to chest X-ray. However, the model’s performance was lower (AUC = 80%) and had many false positives (positive predictive value = 58%), highlighting the need for further improvements. Despite these limitations, LU has the potential to be used by predictive models of ICU admission due to its low-cost, non-ionizing radiative nature, and bedside availability [8]. Other studies have also explored the use of LU to predict the need for ICU admission [9,10]. Alencar et al. [9] and Trias-Sábrai et al. [10] used total LU scores (LUSs) in their analysis, achieving AUCs of 85% and 71.6%, respectively. However, these studies used different scoring methods, a common issue in LU research [1,11,12,13,14]. LU findings (LUFs), such as B-lines, pleural line, or consolidations, are weighted differently across studies, and there is no standardized method for calculating LUS, which may lead to inconsistency in clinical research.

In recent years, machine learning (ML) has shown promise in predicting ICU admission by analyzing datasets that include clinical and laboratory variables [15,16,17,18]. Saadatmand et al. [15] achieved an AUC of 97.6%, but their model relied heavily on synthetic data (88% of ICU cases), which may compromise its robustness. Similary, Altini et al. [16] reported results of AUC = 95.1% but used imprecise time-related variables (such as minimum and maximum CRP values), which complicates its clinical application and did not account for dataset imbalance. Aznar-Gimeno et al. [18] also developed a model for estimating the risk of ICU admission or mortality, with an AUC of 83.1%.

Despite these advances, no studies have combined ML with LU and blood gas analysis (BGA) metrics, such as the P/F ratio, to predict ICU admission. Additionally, while LU has been widely studied, there is no standardized method for extracting information from LU videos.

In this study, we aim to improve ICU admission decisions. To this end, we developed ML models that integrate laboratory findings, including BGA metrics, with LU data to predict the need for ICU admission. We also explored alternative methods for extracting meaningful information from LU videos using ML. Additionally, the results of our model are made available in a web-based app intended for research purposes only (https://predicticu.streamlit.app/ (accessed on 27 January 2025)). This app offers a platform to test the model’s efficacy and improve its predictive capabilities. In the future, we aim to create a prediction tool to be used in clinical practice.

## 2. Materials and Methods

### 2.1. Dataset

The data used in this study were gathered at Hospital Garcia de Orta, Almada, Portugal, and comprised 51 adult patients (45% female; mean age = 62.5 years old) diagnosed with COVID-19, as thoroughly detailed in [1]. The dataset encompasses comprehensive information, including demographic details, past medical history, clinical symptoms and signs, laboratory findings, and treatment records. Additionally, lung point-of-care ultrasound (POCUS) videos were systematically acquired as part of the clinical investigation. The patients were screened in four regions in each hemithorax: two anterior and two lateral regions. Of all the 51 patients, 31 were admitted to the ICU (≈61%).

### 2.2. Lung Ultrasound Scoring Systems

The extraction of information from an LU exam is based on the observation of LUFs, such as B-lines, the pleural line, or consolidations. The combination of these LUFs to calculate an LUS can vary, as described in Section 1. Based on the annotations provided in the dataset for each LU exam, three methods from the literature were considered for LUS calculation: LUS1 [1], LUS2 [13], and LUS3 [14]. Details about the calculation for each score are provided in Table 1.

These three methods assign a weighted score to each LUF and sum them to obtain the total LUS. However, this approach may be influenced by the predefined weights assigned to each LUF, which can depend on user’s experience. To address this, we propose an alternative method for extracting meaningful information from LU videos. Instead of relying on predefined weights, our method extracts individual LUFs separately and allows ML algorithms to determine the optimal combination or weights for each LUF in relation to a specific clinical task—in this case, ICU admission. The extracted LUFs include the Pleura Score, B-lines Score, Subpleural Consolidations (S-Cons) Score, and Lobar (L-Cons) Consolidations Scores.

### 2.3. Selection of Laboratory Parameters

**Figure A1:**
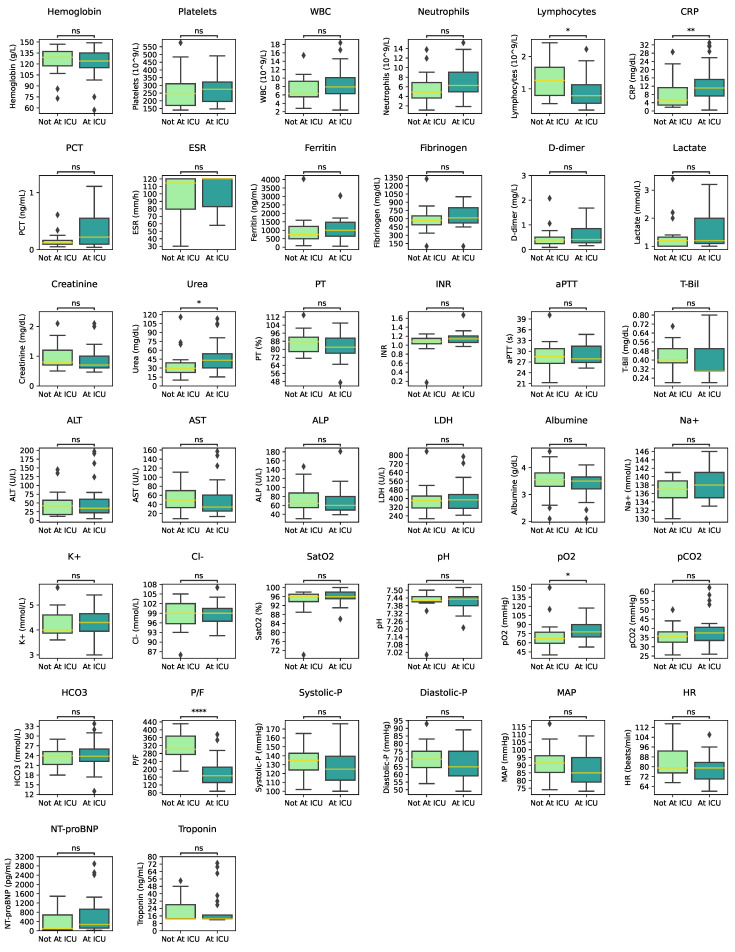
Boxplots of the distribution of every laboratory variable based on ICU admission. Mann–Whitney Test: non-significant (ns): *p* > 0.05; *: 0.01 < *p* ≤ 0.005; **: 0.001 < *p* ≤ 0.01; ****: *p* ≤ 0.0001.

To study the benefits of combining LU information with other laboratory parameters for predicting ICU admission, an exploration of 38 of such parameters was done (see Figure A1 in Appendix A). Mann–Whitney tests were conducted for each parameter as a means for feature selection assessment (α<0.05). Only the significant features were used for building the prediction models in combination with the total LUSs and LUFs.

### 2.4. Prediction Models Development

As stated in Section 1, this study aimed to combine several laboratory findings with LU data for predicting the need for ICU admission based on ML models.

Support vector machine (SVM) was selected as the algorithm of choice after an initial round of testing, where it demonstrated good performance. To mitigate overfitting and provide a more robust analysis, we employed leave-one-out cross-validation, as it is particularly well-suited for small datasets, which is the case of our analysis. Three kernel types were evaluated for SVM: sigmoid, radial basis function (RBF), and linear. For the sigmoid and RBF kernels, both the γ and C hyperparameters were optimized using Bayesian optimization while, for the linear kernel, only the C parameter was optimized. A total of 25 runs of Bayesian optimization were performed to fine-tune the hyperparameters [19]. Additionally, we combined all significant laboratory findings and different LU scoring systems as input features for the SVM model, allowing us to assess the impact of different clinical factors on model accuracy.

**Figure 1:**
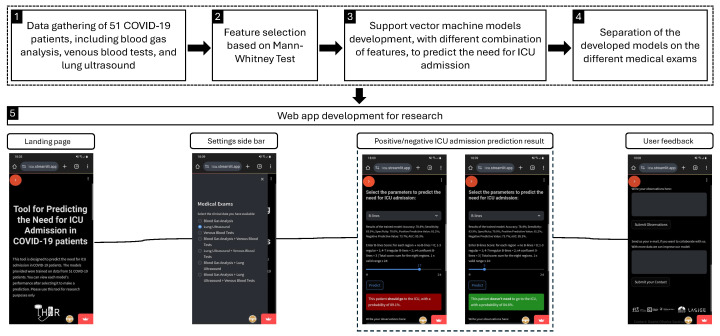
Graphical methodology of the study, presenting each step of the process, from data gathering to the development of the web app.

The results were combined into a web app that is intended for research purposes only. Figure 1 illustrates the overall methodology of the study, presenting each step of the process, from data gathering to the development of the web app.

## 3. Results

### 3.1. Lung Ultrasound Scoring Systems

**Figure 2:**
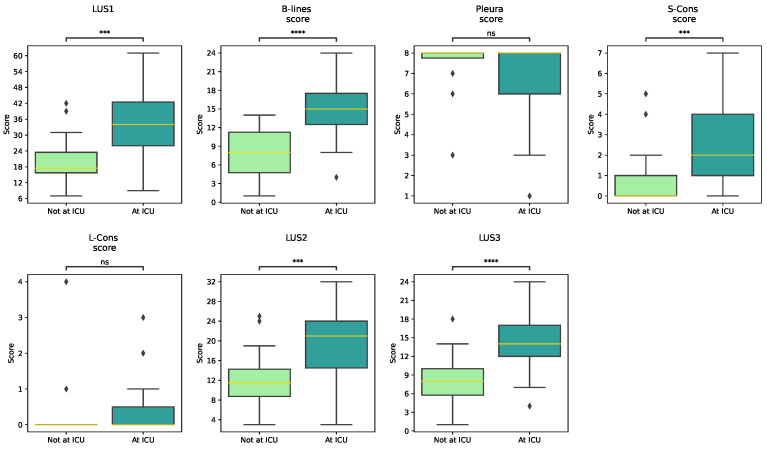
Boxplots of the distribution of all lung ultrasound scoring systems (LUS1; LUS2; LUS3; B-line score; Pleura score; Subpleural Consolidations (S-Cons) score; Lobar Consolidations (L-Cons) score; LUS score 2; LUS score 3)), according to ICU admission. Mann–Whitney Test: non-significant (ns): *p* > 0.05; ***: 0.0001 < *p* ≤ 0.001; ****: *p* ≤ 0.0001.

A statistical analysis of several LU scoring systems was conducted based on ICU admission, and the results are displayed in Figure 2, which displays the boxplots of the distribution of LU scoring systems.

All LU scoring systems were found to be statistically significant in terms of ICU admission (*p* < 0.05), except Pleura score and L-Cons score. Compared with the other LUFs, B-lines seem to be the most significant (*p* < 0.0001).

The different LU scoring systems were also used as input for an ML model to predict the need for ICU admission. LUS1, LUS2, and LUS3 were used individually since they represent total LUS. In contrast, the B-line score, Pleura score, S-Con score, and L-Cons score were fed into the model, both separately and in combination. This approach allowed for the prediction model to determine the optimal combination of these LUFs. Table 2 shows the results for each combination of features.

**Figure 3:**
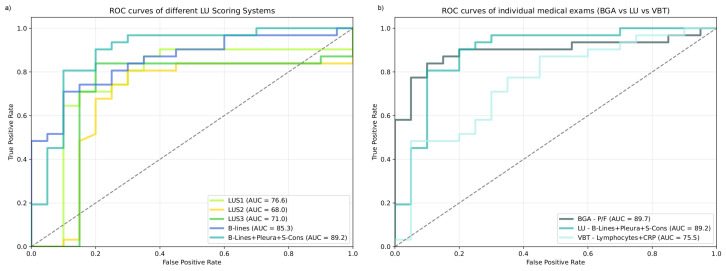
ROC curves of (**a**) the three total lung ultrasound (LU) scores (LUS1, LUS2, and LUS3), B-lines alone, and the best-performing model of LU (B-lines + Pleura + S-Cons), and (**b**) the best-performing models for each medical exam (blood gas analysis (BGA), lung ultrasound (LU), and venous blood tests (VTBs)).

The model combining B-lines, Pleura, and S-Con scores achieved the highest results in terms of accuracy (86.3%), sensitivity (93.5%), specificity (75.0%), positive predictive value (PPV) (85.3%), negative predictive value (NPV) (88.2%), and AUC (89.2%). The model combining Pleura, S-Cons, and L-Cons scores matched these results in sensitivity. Regarding traditional LU scoring systems, the models using LUS1 and LUS3 generally outperformed LUS2. LUS1 and LUS3 achieved identical values across all metrics, except for AUC, where LUS1 obtained a higher result. Among the individual LUFs, B-lines showed the best performance across all metrics. Furthermore, the B-lines model outperformed all total LUS models (LUS1, LUS2, LUS3) across every metric, except sensitivity, where it achieved the same results as LUS1 and LUS3. Overall, models that included B-lines consistently achieved higher AUC than others. Figure 3a displays a comparison between the ROC curves of the three total LU scores, B-lines alone, and the B-lines + Pleura + S-Cons model. The figure further corroborates the findings, demonstrating that the integration of ML substantially improved the AUC and that, with this method, B-lines alone can achieve better performance than the currently used total LUSs.

### 3.2. Laboratory Parameters

**Figure 4:**
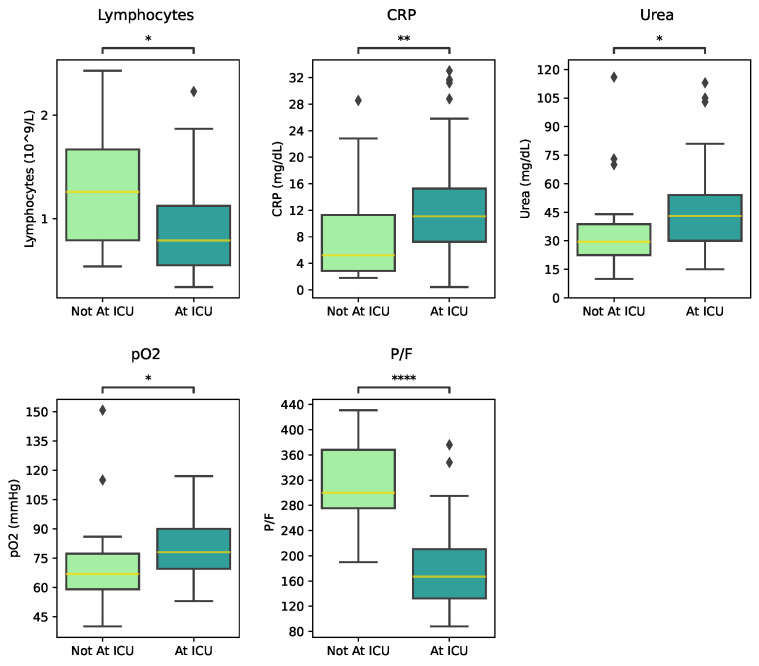
Boxplots of the distribution of lymphocytes, C-reactive protein (CRP), urea, and P/F values, according to ICU admission. Mann–Whitney Test: non-significant (ns): *p* > 0.05; *: 0.01 < *p* ≤ 0.05; **: 0.001 < *p* ≤ 0.01; ****: *p* ≤ 0.0001.

Resulting from feature selection, five laboratory variables were considered significant for building the prediction models: lymphocytes; CRP; urea; ${\mathrm{pO}}_{2}$; and the P/F (Figure 4).

Although both ${\mathrm{pO}}_{2}$ and the P/F ratio were found to be statistically significant, we opted to consider the P/F ratio exclusively in subsequent analyses, as ${\mathrm{pO}}_{2}$ is used to compute the P/F ratio, and including both would introduce redundant features.

From the selected features, lymphocytes, CRP, and urea can all be assessed from venous blood tests (VTBs), whereas the P/F requires arterial blood gas analysis (BGA) to obtain the ${\mathrm{pO}}_{2}$. Table 3 presents the performance of the ICU prediction models separated in VTBs and BGA, as they require completely different procedures.

The model that uses P/F achieves the highest results for every metric when compared with any model derived from VBT parameters. Among the VBT models, only Lymphocytes + CRP and CRP + Urea achieved an AUC greater than 55%, with values of 75.5% and 68.1%, respectively.

Figure 3b displays a comparison between the best-performing models of the different medical exams (BGA, LU, VBT). It is shown that both BGA and LU achieved higher AUC than VTBs, and that their performance is similar, with BGA tending to have better specificity, while LU tends to have better sensitivity.

### 3.3. Combination of Lung Ultrasound, Venous Blood Tests, and Blood Gas Analysis

Another analysis combined features from the different exams (BGA, LU, and VBT) to study the benefits of proceeding with more than one medical exam to predict the need for ICU admission. Considering that this would lead to a very extensive list of results, only the performance metrics of the best models for each combination are shown in Table 4.

The combination of all three exams (BGA + LU + VBT) yields the best results across all metrics. Among the models using two medical exams, the highest AUC is achieved for BGA + LU (AUC = 95.6%), while LU + VBT and BGA + VBT achieve AUCs of 93.1% and 90.8%, respectively.

**Figure 5:**
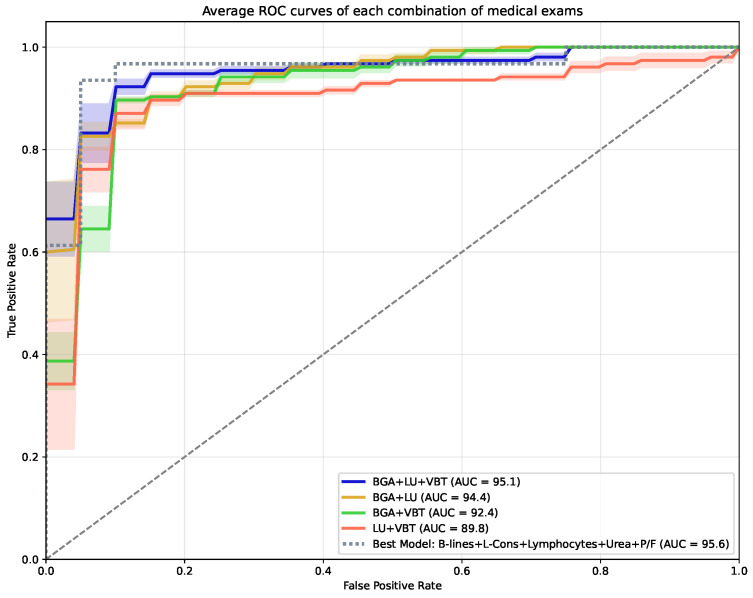
Averaged ROC curve and standard error of the mean (SEM) for each combination of medical exam (blood gas analysis (BGA) + lung ultrasound (LU) + venous blood tests (VTBs), BGA + LU, BGA + VBT, LU + VBT). The shaded regions represent the SEM, highlighting the variability in diagnostic performance. Additionally, the ROC curve of the best-performing model (B-lines + L-Cons + Lymphocytes + Urea + P/F) is included for comparison.

In terms of accuracy (92.2%) and PPV (93.5%), the BGA + VBT model outperforms the best LU + VBT and BGA + LU combinations. Regarding sensitivity, the BGA + LU combination achieve the highest result (96.8%). As for specificity, both BGA + VBT and BGA + LU achieve 90.0%, whereas the best LU + VBT model achieves 85.0%. Figure 5 displays the average ROC curve for the different combinations of medical exams (BGA + LU + VBT, BGA + LU, BGA + VBT, LU + VBT). For comparison, the ROC curve of the best-performing model in terms of accuracy, sensitivity, specificity, PPV, and NPV (B-lines + L-Cons + Lymphocytes + Urea + P/F) is also displayed. In terms of average AUC, it is shown that the combination of the three medical exams (BGA + LU + VBT) yields the highest results (AUC = 95.1%), sequentially followed by BGA + LU (AUC = 94.4%), BGA + VBT (AUC = 92.4%), and LU + VBT (AUC = 89.8%), respectively.

Finally, the results here presented are publicly available for research purposes in a web app developed by the authors. The link is https://predicticu.streamlit.app/ ((accessed on 27 January 2025) see Appendix A.2 in Appendix A).

## 4. Discussion

### 4.1. Lung Ultrasound Scoring Systems

Considering the statistical analysis of the different LU scoring systems, it was shown that B-line and S-Con scores were significant in determining if a patient should or should not be admitted to the ICU. This was not true for Pleura or L-Con scores. These results are also supported by the developed prediction models, where Pleura and L-Con scores alone only obtained AUCs of 24.0% and 26.3%, respectively. On the contrary, B-lines were clearly the LUF that led to the best results, as the B-line score model outperformed all individual LUF models in every performance metric. B-lines alone also achieved higher results in all metrics compared to LUS1, LUS2, and LUS3, except for sensitivity, where the results were equal. This shows that the sole observation of B-lines and posterior ML processing may be enough to identify patients’ outcomes better than the total LUS usually reported in the literature. Additionally, models that included the B-line score consistently achieved higher AUC results than those that did not use B-lines as an input feature. This is a noteworthy result, as AUC is a probabilistic measure of the classifier’s ability to accurately distinguish between the two classes. It may better reflect the model’s robustness and efficacy compared to the other performance metrics.

This analysis also allows us to compare the LUSs reported in the literature with an alternative method introduced here for extracting information from LU data. This new approach involved finding the optimal combination of different LUFs for an ML task.

The results demonstrate that the alternative method can produce better models, with the highest AUC reaching 89.2% for the combination of B-lines, Pleura, and S-Cons, compared to the best AUC of 76.6% from models based on total LUS. A similar pattern is observed in terms of sensitivity (93.5% vs. 83.9%), specificity (75.0% vs. 65.0%), PPV (85.3% vs. 78.8%), and NPV (88.2% vs. 72.2%). The results show that the combination of individual LUFs with ML yields better predictions for ICU admission compared to LUS1, LUS2, and LUS3, which are commonly used in clinical settings. Furthermore, compared with the literature, the AUC in our study (89.2%) is also higher than the AUCs reported in previous studies that focused on computing a total LUS: 85% [9] and 71.7% [10].

Therefore, the results of our study suggest that the reliance on total LUSs, which are calculated by attributing predefined weights to each LUF, may not extract the maximum potential of a nLU exam. As such, this study emphasizes the need for an ML approach that mathematically combines the individual LUFs to enhance predictions of clinical outcomes.

### 4.2. Prediction of Intensive Care Unit Admission

#### 4.2.1. Comparison Between the Different Medical Exams

Considering the results in Table 2 and Table 3 and Figure 3, we can compare the three different medical exams explored in this study. The findings indicate that the BGA model and the best LU model (B-lines + Pleura + S-Cons) outperform the best VBT model across all metrics.

When comparing the performance metrics of the best LU model and the BGA model, we observe differences of 0.0%, +3.2%, −5.0%, −2.2%, +4%, and −0.5%, for accuracy, sensitivity, specificity, PPV, NPV, and AUC, respectively. In a clinical setting, sensitivity and NPV are often prioritized over specificity and PPV, as they directly address the need to minimize false negatives. Failing to identify a patient who requires ICU care could have life-threatening consequences, making it relevant to focus on sensitivity and NPV. In this context, the LU model is more effective at reducing false negatives, while the BGA model is better at minimizing false positives. The magnitude of improvement in reducing false negatives with the LU model is comparable to the reduction in false positives achieved by the BGA model. Additionally, the BGA model leads to a slight increase in AUC. Given these trade-offs, the choice on which model to use may depend on various factors, including the relative importance of false negatives versus false positives and overall performance metrics. Considering this, both medical exams demonstrate excellent performance in predicting the need for ICU admission and can be effectively used in practice. Additionally, our analysis shows that the single use of either LU or BGA surpasses the performance of the GRAM [6] and COWS [7] scores, which rely on clinical history and more clinical parameters. This underscores the value of ML models in enhancing predictions for the need of ICU admission, offering greater precision and efficiency with less patient information required.

#### 4.2.2. Comparison Between the Combination of the Different Medical Exams

The combination of features from the different exams (BGA, LU, and VBT) (Table 4 and Figure 5) generally results in better prediction for the need of ICU admission than the single use of either BGA, LU, or VBT (Table 2 and Table 3 and Figure 3). However, both the best LUF model and the P/F model still obtain results comparable with some of the models presented in Table 4. Therefore, the inclusion of more than one medical exam may improve the prediction, but careful considerations should be made regarding the available resources. For the combination of two medical exams, the combination of BGA and LU achieves the highest AUC (95.6%), whereas the combination of LU and VBT, and BGA and VBT achieves AUCs of 93.1% and 90.8%, respectively. This supports the idea that if two medical exams are being performed, both LU and BGA should be prioritized. Nonetheless, this depends on several factors, including the available resources and the performance metrics that are considered more relevant. In cases where the priority is given to reducing the number of false negatives, combining BGA with LU seems to be very important, as sensitivity and NPV are substantially higher compared with the models that include VTBs. In terms of false positives, it seems to be essential to use P/F in the model, as the best model that does not include P/F (LU + VBT) achieves less than 5% of specificity and less than 3.2% of PPV compared with BGA + LU and BGA + VBT. This is relevant for avoiding spending resources on patients who would not need ICU treatment. Finally, if AUC is considered the most relevant metric, the inclusion of LU consistently improves the results. Overall, although there seems to be a general superiority of the BGA + LU model, as it seems to create the best balance across the different metrics, the two other options present models that seem to achieve similar performances.

Moreover, this study shows that using the three medical exams leads to the best results across all metrics. However, different models produce varying outcomes. As an example, the B-lines + Pleura + S-Cons + Lymphocytes + CRP + Urea + P/F model should be the model to use when prioritizing AUC. On the contrary, if the reduction of both false negatives and false positives is more relevant, B-lines + L-Cons + Lymphocytes + Urea + P/F should be the model to use. Overall, we demonstrate that there are different models that perform well, depending on the resources available at the hospital. These models can be tested in our web app. The choice to include several exams in the analysis may thus hinge on the specific emphasis placed on each performance metric, as well as on the available resources, highlighting the importance of tailoring the model to the desired clinical outcome.

Compared with similar studies in the literature that aim to predict ICU admission using ML, we achieved satisfactory and promising results. Nonetheless, care should be taken in such comparisons, as the data and methodology used in the different studies are different. As previously mentioned, the best model always depends on the preferred clinical outcomes. For comparison with other studies, although the model with the highest AUC achieves 96.5%, we consider the best model to be the one incorporating B-lines, L-Cons, Lymphocytes, Urea, and the P/F ratio. This model achieves the highest accuracy, sensitivity, specificity, PPV, and NPV while still maintaining a high AUC of 95.5%.

In terms of AUC, our model (AUC = 95.5%) is outperformed by the best model of the study of Saadatmand et al. [15] (AUC = 97.6%). However, the studies of [16,18] achieved AUCs of 83.1% and 95.1%, respectively. Regarding sensitivity, the authors of [15,16,18] achieved values of 91%, 67%, and 71%, respectively. Our model outperformed these studies, as the sensitivity was 96.8%. Regarding specificity, our model obtains values of 90.0%, which is lower than that of [15] (96%), but outperforms the results of [18] (78%). In terms of PPV, our model obtained a value of 93.8%, being outperformed by [15] (97%), and surpassing [16] (91%) and [18] (60%). For NPV, [15,18] obtained results of 89% and 85%. Our model achieved a better result (94.7%). In terms of accuracy, [15,16,18] obtained values of 93%, 90%, and 76%. Our best model achieved an accuracy of 94.1%, which outperforms the three studies. In general, our best model outperforms the studies mentioned here that used ML to predict ICU admission. The same applies to the studies mentioned in Section 1 that used LU [9,10]. Overall, this study reinforces the pivotal role of ML models in predicting ICU admission and, in particular, the advantages of including LU exams in this clinical decision.

Despite the study’s contributions, it is essential to acknowledge its limitations. First, while we evaluated different SVM kernels, we did not perform an extensive comparison with other ML algorithms. Future exploration of models such as gradient boosting machines or neural networks could potentially yield improved performance. Additionally, the small sample size poses significant challenges in developing a reliable and widely applicable ML model. Although the number of features was small and a leave-one-out approach was applied, the limited sample size may lead to high variance in performance estimates, making it harder to generalize to new data.

Moreover, the study used data from a single hospital (Hospital Garcia de Orta), which may limit the model’s generalizability to other healthcare settings. Hospital Garcia de Orta is a specialized medical center with advanced diagnostic and treatment facilities. These characteristics may differ from hospitals with fewer resources, potentially impacting the applicability of our findings. Furthermore, clinical practices and ICU admission criteria may vary between hospitals, affecting external validity. Factors such as hospital capacity and patient volume, which fluctuated significantly during the COVID-19 pandemic—the period of data collection—could also influence admission decisions. In future work, incorporating data from additional hospitals with varying resources, patient demographics, and clinical workflows would improve the generalizability of our web app and mitigate potential biases.

Nonetheless, our findings demonstrate that LU is a valuable tool for predicting ICU admission. Compared to BGA, LU appears to produce fewer false negatives, which is critical in this clinical setting. This study primarily focused on evaluating the impact of different data inputs (LU, BGA, and VBT) on predicting ICU admission and on exploring various methods for extracting meaningful information from LU. These findings provide valuable insights into the potential of ML models for ICU admission prediction. However, creating a broadly generalizable model was beyond the scope of this initial study.

Future studies should focus on developing a more generalizable ML model by utilizing larger, more diverse datasets and incorporating external validation sets to evaluate model performance across varying clinical contexts. These efforts would enhance the applicability of the proposed methodology and further validate its potential to improve clinical decision-making.

## 5. Conclusions

This study demonstrates that there is no standardized LUS that extracts the maximum information from LU exams. Instead, ML emerges as a powerful tool capable of leveraging various LUFs to predict specific disease outcomes, thereby supporting more informed and individualized clinical decisions. This study argues against the necessity of calculating a total LUS unless it directly contributes to optimal patient follow-up. In this study, B-lines alone achieved higher AUCs than the LUS reported in the literature.

Furthermore, the study underscores the comparability of LU with P/F, a widely recognized metric, and emphasizes LU’s superiority in reducing the number of false negatives, an essential factor for critical clinical decisions such as ICU admission.

Given the lack of a standardized method for predicting ICU admission, this study supports the potential benefits of using an ML tool that integrates LUFs with laboratory variables. This approach has been translated into a web application, offering a practical implementation of the model. Our best model is comparable to others in the literature and outperforms them in terms of sensitivity and NPV, which are considered essential for the clinical decision of ICU admission.

Future research should focus on larger and more diverse patient cohorts to enhance the model’s generalizability and assess additional outcomes, such as mortality. Moreover, automating the extraction of LUFs could improve the application of this methodology in clinical practice.

In conclusion, this research not only offers valuable insights into the predictive power of specific medical exams but also underscores the potential of ML in combining multiple medical exams to improve overall performance and support clinical decision-making. 

## Figures and Tables

**Table 1:** Lung ultrasound scoring Systems. LUS1 [1], LUS2 [13], and LUS3 [14], for which scores are assigned based on specific artifact findings (Pleura, B-lines, and Consolidations). The total score for a patient corresponds to the sum of all artifact scores across the eight lung regions. The total possible scores for LUS1, LUS2, and LUS3 range from 0 to 104, 0 to 32, and 0 to 24, respectively. Regarding lung ultrasound findings (LUFs)—Pleura Score, B-lines Score, Subpleural Consolidations (S-Cons) Score, and Lobar Consolidations (L-Cons) Score)—each type of artifact finding is scored separately. Each LUF is scored by summing its values across the eight regions.

LUS1									
Pleura		B-lines				Consolidations			Total
Normal	Irregular	0	1–3	4–7	≥4 confluent	0	≥1	≥1	Sum of
		B-lines	B-lines	B-lines	B-lines	Consolidations	S-Cons	L-Cons	All
0	1	0	1	2	3	0	4	5	Scores
**LUS2**									
Pleura		**B-lines and Consolidations**						**Total**	
Normal	Irregular	≤3 B-lines	>3 B-lines	≥4 confluent B-lines		≥1 consolidation		Sum of	
0	1	0	1	2		3		All Scores	
**LUS3**									
**Pleura, B-lines, and Consolidations**							**Total**		
Normal	≤1 B-lines	>1 confluent B-lines	≥1 consolidation + normal pleura		≥1 consolidation + normal pleura		Sum of		
0	1	2	2		3		All Scores		
**LUF**									
**Pleura Score**		**B-lines Score**				**S-Cons Score**		**L-Cons Score**	
Normal	Irregular	No B-lines	1–3 B-lines	4–7 B-lines	≥4 confluent	No	≥1	No	≥1
					B-lines	S-Cons	S-Cons	L-Cons	L-Cons
0	1	0	1	2	3	0	1	0	1

**Table 2:** Accuracy (Acc), sensitivity, specificity, positive predictive value (PPV), negative predictive value (NPV), and area under the receiver operating (ROC) curve (AUC) for the different combinations of lung ultrasound (LU) variables. The highest values for each metric are bold.

Features	Acc	Sensitivity	Specificity	PPV	NPV	AUC
LUS1	76.5	83.9	65.0	78.8	72.2	76.6
LUS2	74.5	80.6	65.0	78.1	68.4	68.0
LUS3	76.5	83.9	65.0	78.8	72.2	71.0
B-lines	78.4	83.9	70.0	81.2	73.7	85.3
Pleura	58.8	96.8	0.0	60.0	0.0	24.0
S-Cons	64.7	83.9	35.0	66.7	58.3	64.0
L-Cons	58.8	96.8	0.0	60.0	0.0	26.3
B-Lines + Pleura	78.4	87.1	65.0	79.4	76.5	87.4
B-lines + S-Cons	78.4	87.1	65.0	79.4	76.5	82.3
B-lines + L-Cons	74.5	83.9	60.0	76.5	70.6	80.2
Pleura + S-Cons	78.4	83.9	70.0	81.2	73.7	74.8
Pleura + L-Cons	60.8	100.0	0.0	60.8	0.0	33.0
S-Cons + L-Cons	72.5	87.1	50.0	73.0	71.4	67.4
B-Lines + Pleura + S-Cons	**86.3**	**93.5**	**75.0**	**85.3**	**88.2**	**89.2**
B-lines + Pleura + L-Cons	74.5	90.3	50.0	73.7	76.9	87.9
B-lines + S-Cons + L-Cons	76.5	87.1	60.0	77.1	75.0	84.0
Pleura + S-Cons + L-Cons	80.4	**93.5**	60.0	78.4	85.7	75.9
B-Lines + Pleura + S-Cons + L-Cons	82.4	90.3	70.0	82.4	82.4	86.9

**Table 3:** Accuracy (Acc), sensitivity, specificity, positive predictive value (PPV), negative predictive value (NPV), and area under the receiver operating characteristic (ROC) curve (AUC) for variables related to venous blood tests (VTBs) and blood gas analysis (BGA). The highest values for each metric are bold.

Features	Acc	Sensitivity	Specificity	PPV	NPV	AUC
Venous Blood Tests						
Lymphocytes	56.9	90.3	5.0	59.6	25.0	39.7
CRP	64.7	90.3	25.0	65.1	62.5	55.0
Urea	52.9	83.9	5.0	57.8	16.7	33.1
Lymphocytes + CRP	68.6	87.1	40.0	69.2	66.7	75.5
Lymphocytes + Urea	51.0	83.9	0.0	56.5	0.0	35.6
CRP + Urea	64.7	80.6	40.0	67.6	57.1	68.1
Lymphocytes + CRP + Urea	51.0	83.9	0.0	56.5	0.0	49.1
Blood Gas Analysis						
P/F	**86.3**	**90.3**	**80.0**	**87.5**	**84.2**	**89.7**

**Table 4:** Accuracy (Acc), sensitivity, specificity, positive predictive value (PPV), negative predictive value (NPV), area under the receiver operating characteristic (ROC) curve (AUC) for the different combinations of features from different medical exams: blood gas analysis (BGA), lung ultrasound (LU)), and venous blood tests (VTBs). The highest values for each metric are bold.

Features	Acc	Sensitivity	Specificity	PPV	NPV	AUC
Blood Gas Analysis + Venous Blood Tests						
Lymphocytes + CRP + P/F	82.4	90.3	70.0	82.4	82.4	90.8
CRP + Urea + P/F	84.3	90.3	75.0	84.8	83.3	90.6
Lymphocytes + CRP + Urea + P/F	82.4	90.3	70.0	82.4	82.4	90.3
Lymphocytes + Urea + P/F	92.2	93.5	**90.0**	93.5	90.0	88.4
Lymphocytes + P/F	88.2	90.3	85.0	90.3	85.0	87.4
Lung Ultrasound + Venous Blood Tests						
B-lines + Pleura + S-Cons + Lymphocytes + CRP + Urea	88.2	90.3	85.0	90.3	85.0	93.1
B-lines + Pleura + S-Cons + L-Cons + Lymphocytes + Urea	88.2	90.3	85.0	90.3	85.0	92.9
B-lines + Pleura + Lymphocytes + CRP + Urea	86.3	90.3	80.0	87.5	84.2	92.4
B-lines + Pleura + Lymphocytes + CRP	86.3	90.3	80.0	87.5	84.2	91.8
B-lines + Pleura + S-Cons + Lymphocytes	88.2	90.3	85.0	90.3	85.0	90.8
Blood Gas Analysis + Lung Ultrasound						
B-lines + Pleura + S-Cons + P/F	88.2	90.3	85.0	90.3	85.0	95.6
B-lines + Pleura + L-Cons + P/F	88.2	87.1	**90.0**	93.1	81.8	95.0
B-lines + P/F	86.3	90.3	80.0	87.5	84.2	94.0
B-lines + S-Cons + P/F	88.2	90.3	85.0	90.3	85.0	94.0
B-lines + Pleura + P/F	86.3	**96.8**	70.0	83.3	93.3	92.6
Blood Gas Analysis + Lung Ultrasound + Venous Blood Tests						
B-lines + Pleura + S-Cons + Lymphocytes + CRP + Urea + P/F	90.2	93.5	85.0	90.6	89.5	**96.5**
B-lines + L-Cons + Lymphocytes + CRP + Urea + P/F	90.2	93.5	85.0	90.6	89.5	95.6
B-lines + L-Cons + Lymphocytes + Urea + P/F	**94.1**	**96.8**	**90.0**	**93.8**	**94.7**	95.6
B-lines + Pleura + L-Cons + Lymphocytes + Urea + P/F	90.2	93.5	85.0	90.6	89.5	95.5
Pleura + Lymphocytes + CRP + Urea + P/F	88.2	93.5	80.0	87.9	88.9	91.8

**Figure A2:**
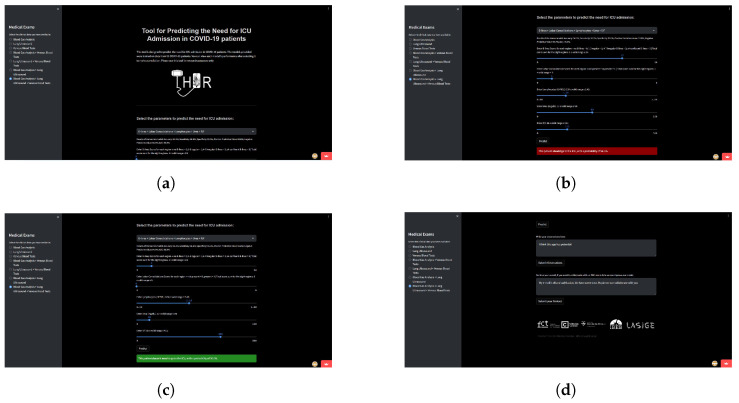
User guide for using the web app. (**a**) 1°—Select a possible combination of medical exams; (**b**) 2°—Based on the input values, this patient should go to the ICU; (**c**) 3°—Based on the input values, this patient should not go to the ICU; (**d**) 4°—Here, you can send some observations and feedback related to your experience using the web app.

**Figure A3:**
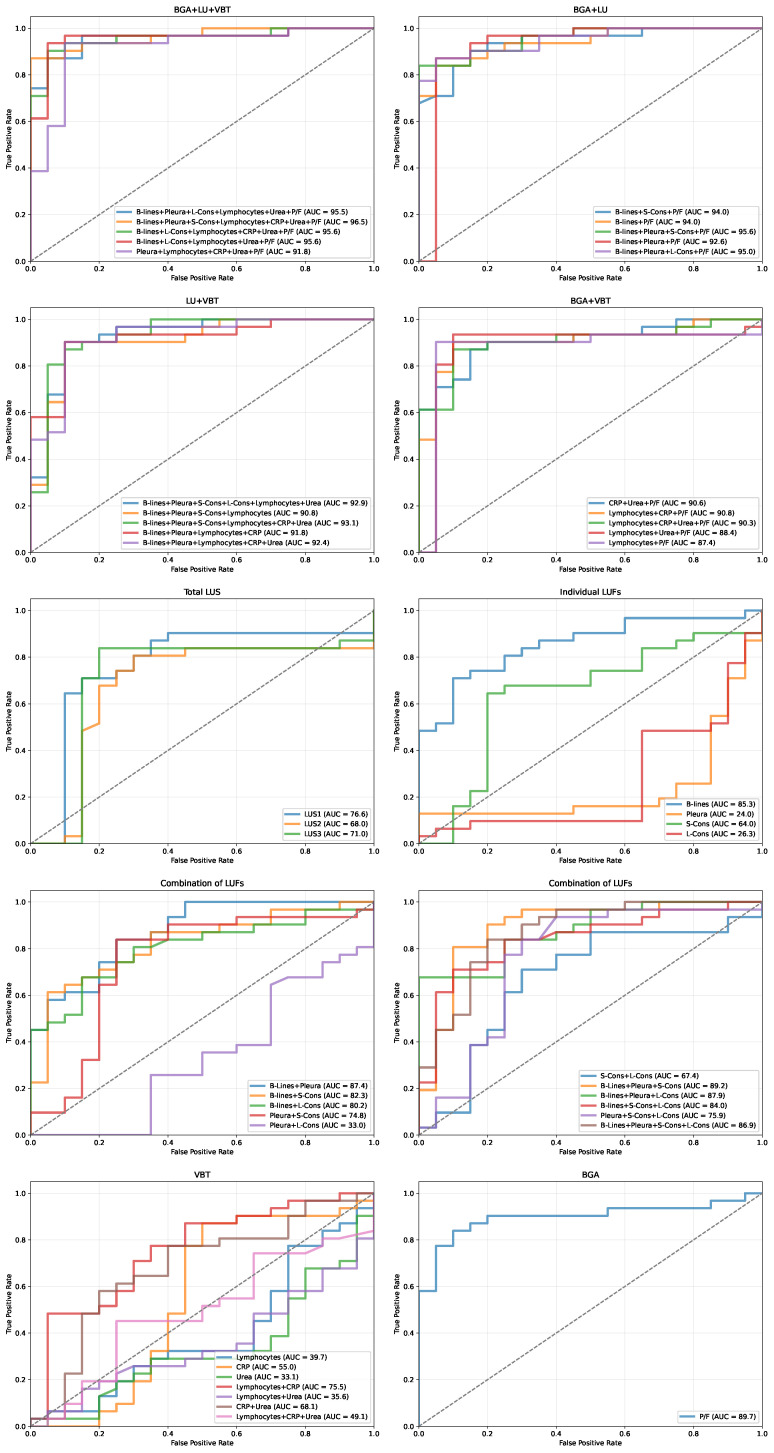
ROC curves of all models.

## Data Availability

Data were obtained at Hospital Garcia de Orta and is not publicly available.

## Appendix A

### Appendix A.1. Laboratory Parameters

The distribution of the 38 laboratory parameters in terms of ICU admission is shown in Figure A1: hemoglobin; platelets; white blood cells (WBC); neutrophils; lymphocytes; C-reactive protein (CRP); procalcitonin (PCT); erythrocyte sedimentation rate (ESR); ferritin; fibrinogen; D-dimer; lactate; creatinine; urea; prothrombine time (PT); international normalized ratio (INR); activated partial thromboplastin time (aPTT); total bilirubin (T-Bil); alanine aminotransferase (ALT); aspartate aminotransferase (AST); alkaline phosphatase (ALP); lactate dehydrogenase (LDH); albumine; sodium (${\mathrm{Na}}^{+}$); potassium ($K^{+}$); chloride (${\mathrm{Cl}}^{-}$); saturation of oxygen (${\mathrm{SatO}}_{2}$); pH; arterial partial pressure of oxygen (${\mathrm{pO}}_{2}$); arterial partial pressure of carbon dioxide (${\mathrm{pCO}}_{2}$); bicarbonate (${\mathrm{HCO}}_{3}$); ratio between the ${\mathrm{pO}}_{2}$ and fraction of inspired oxygen (P/F ratio); systolic pressure; diastolic pressure; mean arterial pressure (MAP); heart rate (HR); N-terminal pro–B-type natriuretic peptide (NT-proBNP); troponin.

Mann–Whitney tests were applied to every parameter to assess significant differences between patients admitted or not to the ICU (α<0.05).

### Appendix A.2. Web App

The web app here developed is intended for research purposes only. It is available through the following link: https://predicticu.streamlit.app/ (accessed on 27 January 2025).

If, when opening the web app, the following message appears: “This app has gone to sleep due to inactivity. Would you like to get it back up?”, please press the “Yes, get this back up!” button.

The web app is organized based on the different medical exams that the user may have available. For each type of medical exam (BGA, LU, and VBT), there are several ML models that you may use to attempt to obtain the prediction.

#### Appendix A.2.1. Illustrative Example

In this example, we focused on the model that was considered the best performer (B-lines + L-Cons + Lymphocytes + Urea + P/F). First, we selected a possible combination of medical exams. In this example, we decided to combine all medical exams available (BGA, LU, and VBT). Next, we defined the model that would be used for prediction, which included the following clinical features: B-lines, L-Cons, Lymphocytes, Urea, and P/F. We present two possible clinical scenarios for illustration (Figure A2a). In the first scenario (Figure A2b), high values of B-lines, L-Cons, and Urea, combined with low values of Lymphocytes and P/F ratio were selected. Considering clinical evidence and the patterns observed in the boxplot figures (Figure A1 and Figure 4), it would be expected that this patient would need to go to the ICU. The model suggested that “This patient should go to the ICU, with a probability of 94.3%”, which is in accordance with our assumptions. In the second scenario (Figure A2c), values associated with lower ICU necessity, based on the boxplots (Figure A1 and Figure 4) and clinical evidence, were selected. The model suggested that “This patient doesn’t need to go to the ICU, with a probability of 98.0%”, which is also in line with our assumptions. Finally, the user is invited to provide feedback or make observations about the web app. Additionally, it is encouraged that the user send a contact email to explore potential collaborations, particularly in sharing more data to improve the model (Figure A2d).

### Appendix A.3. ROC Curves of All Models

Figure A3 displays the ROC curves of all models.
